# A Lecture on the Tubercle-Bacillus and Phthisis

**Published:** 1883-12

**Authors:** T. Henry Green

**Affiliations:** Physician to the Hospital, and Senior Assistant-Physician to the Hospital for Consumption and Diseases of the Chest, Brompton


### Selections.

---

A LECTURE ON THE TUBERCLE-BACILLUS AND PHTHISIS. Delivered at Charing Cross Hospital by T. HENRY GREEN, M.D., F.R.C.P., Physician to the Hospital, and Senior Assistant-Physician to the Hospital for Consumption and Diseases of the Chest, Brompton.

GENTLEMEN: The discovery by Dr. Koch of the organism concerned in the production of tuberculous diseases, seems destined to exercise a more important influence upon practical medicine than has any previous outcome of pathological research. This influence is already marked, and although the discovery is but a few months old, both the profession and the public are recognizing its importance, and are actively engaged, by means of respirators and other weapons, in combating the bacillus to which Koch has introduced us. Now, gentlemen, the announcement of any new and great discovery in medical science is apt to unduly divert us from those which have preceded it; and we are, perhaps, rather liable to allow our opinions to be influenced by the new, to the exclusion of the old. This danger I cannot help thinking we are beginning to incur in connection with the tubercle-bacillus. It is not my purpose now to describe to you Koch's all-important investigations, neither to venture to criticise the inferences which have been drawn from them. In dealing with such complex pathological problems, investigations require to be repeated before the results obtained from them can receive unconditional acceptance. I shall, however, to-day, assume the truth of the proposition that tuberculous processes are infective in their nature, and are due to the presence of a specific organism—the bacillus of

Koch; and from this standpoint I wish to consider the subject more especially as it concerns pulmonary phthisis; and I shall endeavor to place before you as briefly as I can, a few considerations which appear to me to be of some practical importance in this connection.

In the first place, then, let me ask you to bear in mind what has been the tendency of pathological teaching respecting phthisis during the past few years. This is, I think, fairly represented in some propositions which I submitted to you when speaking upon this subject more than a year ago. Amongst these you will find the following: 1. The morbid processes which lead to phthisical consolidation of the lung are inflammatory in their nature; by which it is simply meant to imply that they owe their origin to some kind of injury of the pulmonary tissues. 2. The injury of the lungs which leads to phthisis is mainly inflicted through the medium of the bronchi, and consists in some condition of the respired air. 3. It becomes *increasingly probable* that this injurious influence of the respired air is due to the presence in it of *minute organisms;* although whether these organisms are specific, and whether they are themselves the causes of the phthisical process, is at present quite uncertain. 4. In most cases phthisical consolidation of the lung differs from other forms of pneumonic consolidation, inasmuch as it exercises an injurious influence upon the adjacent and distant pulmonary tissues, and thus tends to spread. To this *infective* property, which varies in degree very considerably in different cases, and under different circumstances, the progressive character of phthisical consolidation is largely due.

You see, therefore, that our previous notions of phthisis have, so to speak, prepared us for the discovery of Koch. The tubercle-bacillus is the element that was wanting to establish the truth of our pathology as far as it had gone. But Koch's investigations do much more than this; not only do they show that the infective nature of tuberculous processes is due to organisms, but they appear to prove that these organisms are specific—that tuberculosis is produced by Koch's bacillus, and originates in no other way. This is, undoubtedly, by far the most important advance that has yet been made in the pathology of those diseases. But even now our knowledge is quite incomplete. We have yet to learn what conditions are necessary in order that the bacillus may exercise its injurious influence; for it must, I think, be admitted that something more is required than the mere presence of the organisms.

The mode by which the bacillus obtains an entrance to the lungs is undoubtedly, in all but quite exceptional cases, by means of the respired air. The sputum of phthisical patients abounds in the organisms which, as Koch has shown, retain their vitality after the sputum has dried up, and thus becomes capable of dissemination as dust in the atmosphere; they are, perhaps, also contained in the breath. The source of infection must thus be very generally present. In some situations, as in institutions set apart for the treatment of consumptives, the atmosphere must probably be impregnated with the contagium, and yet how exceedingly rare are instances in which it can be shown that the disease has been communicated. Although isolated cases of probable infection have been met with by Dr. Hermann Weber and others, the evidence of clinical experience is altogether against the contagious nature of phthisis. Notwithstanding, Koch's investigations appear to prove that it is a truly contagious, *i. e.*, communicable disease. It is, however, certainly communicable only under conditions which comparatively rarely obtain. A knowledge of the conditions which are necessary in order that the tubercle-bacillus may produce tuberculosis, is therefore what is now to be desired. We want to know the life history of the contagium. This knowledge must undoubtedly await further experimental research; but in endeavoring to obtain it, I believe we shall again do well to consider some of our old-fashioned notions of pulmonary pathology.

In considering what conditions are necessary in order that the respired bacillus may produce phthisis, I would say, in the first place, that it is probable that this result is in no way influenced by the possibility, or otherwise, of the organisms obtaining a free access to the pulmonary tissues. The bacilli are so minute that they must readily enter the alveoli, and probably also, like the particles of inhaled carbon which give rise to the normal pigmentation of the lungs, be able to penetrate the pseudostomata of Klein, and thus get into the lymph-channels. We must look, therefore, to the pulmonary tissues with which the organisms come into contact. Some abnormal condition of the lung would appear to be a necessary factor in the process. Here there are, I think, two well known elements in the pathology of phthisis, a consideration of which will help us in our inquiry—I mean the apicial distribution of the pulmonary lesion, and the influence of inherited predisposition.

The apicial distribution of the pulmonary lesion would appear alone to be sufficient to show that something more is necessary than the mere presence of the organisms. The causes of this distribution are, as you are aware, to be sought for in the diminished range of respiratory movement which obtains in the highest portions of the lungs. As the result of this diminished movement there is, in certain conditions of health, a tendency to stagnation of the blood-stream in the pulmonary capillaries. The stagnation of the circulation leads to more or less injury of the walls of the pulmonary vessels; the injured vessels are no longer capable of completely retaining the elements of the blood, and the slight leakage thus induced favors active changes in the alveolar epithelium. We have, therefore, in certain individuals, and under certain circumstances, this tendency to slight congestion and inflammation in the highest portions of the lungs; and this tendency appears not only to determine the apicial distribution of the pulmonary consolidation, but its outcome—the presence of inflammatory products, however small in amount, *probably* furnishes one of the conditions necessary in order that the bacilli may cause phthisical disease.

The influence of inherited predisposition is so marked, that its consideration must necessarily occupy a prominent place in any study of the pathology of phthisis. As to the nature of what is transmitted—although, accepting Koch's conclusions, this may be in quite exceptional cases a specific organism or virus, as in the case of syphilis—speaking generally, it is in all probability simply a tendency to disease. Without discussing this subject it may, I believe, be said that the tendency consists in some feebleness of the constitution in general, and often of the lungs and other organs in particular. As a result of this feebleness there is usually a want of constitutional vigor; the power of resisting injurious influences is diminished, and the lungs, and often other organs and tissues, which are especially weak, are, as a consequence, abnormally liable to become inflamed. Further—this inherited weakness not only renders certain organs abnormally liable to inflammation, but also abnormally incapable of recovering from the effects of the inflammatory process; and thus there is more or less tendency to a retention and accumulation of the inflammatory products in the affected area. This tendency to accumulation and infiltration is, as you know, the most important histological characteristic of scrofulous inflammations.

You see, therefore, that the result of any inherited tendency to phthisis must necessarily be to favor the occurrence of those congestive and inflammatory conditions in the apices of the lungs, the existence of which, as we have said, probably constitutes an important factor in the pathological history of the bacillus. General feebleness and want of vigor lead to loss of muscular strength and weakness of the heart, and thus tend to prevent the full expansion of the chest, to cause a stooping posture of the body, and to impair the force of the circulation—all conditions favoring blood-stagnation in the highest portions of the lungs. Further, the toneless condition of the blood-vessels and the poverty of the blood with which the constitutional feebleness is so often associated, furnish the conditions which are the most fovorable to transudation.

Lastly, in connection with this part of our subject I must ask you to bear in mind that all important factor in the pathology of phthisis—the state of the general health. Quite apart from any inherited constitutional feebleness there can be no doubt that an impaired state of health very greatly favors the development of the disease. This fact gives additional support to the view we have been taking, inasmuch as any acquired weakness must necessarily play the same *rôle* in the causation of apicial stagnation and exudation as the inherited feebleness to which we have alluded. It is when the two are associated, as they so frequently are, that we have the conditions most favorable to the development of the disease.

We are now in a position to return to our original question—

what conditions are necessary in order that the respired bacillus may produce phthisis? It seems *probable*, I think, from this brief survey of our old pathology that one of these conditions, at all events, is the presence of some inflammatory products within the pulmonary alveoli. Whether such products favor the growth of the organisms, or whether the presence of the organisms leads to the development of some infective substance in the products, I shall not attempt to discuss. The answer to such and many other questions must, as I have stated, await further investigation. I would, however, here remark on the fact that it is alveolar and not bronchial inflammation which especially favors the development of phthisis; and it is interesting to note in this connection that Koch's investigations show the necessity of *prolonged* contact of the bacilli in order to produce tuberculosis. Such prolonged contact is obviously much more likely to occur in the air-vesicles than in the bronchial tubes.

It appears, therefore, from the present position of our knowledge respecting the pathology of phthisis—accepting the truth of Koch's investigations—that two conditions, at all events, are necessary in order to produce the disease: the presence of the tubercle bacillus, and some abnormal state of the pulmonary tissue with which it comes into contact—an abnormal state obtaining in the very great majority of cases in the highest portions of the lungs, and probably depending, for the most part, upon inherited or acquired constitutional feebleness. Such being the case, we have now to consider how far this teaching ought to influence us in our practice. To what extent is Koch's discovery likely to aid us in the diagnosis and prognosis of the disease? and, above all, what indications does it afford with reference to its treatment? The answer to such questions must necessarily await prolonged and careful clinical study. At present it is impossible to do much more than make suggestions on these heads.

*Diagnosis.*—The tubercle-bacillus is to be found in phthisical sputum. An examination of the sputum may consequently prove a valuable aid to diagnosis. Thus far, the investigations of various observers appear to show that the organisms exist in all cases of this disease. This is important, as it tends to support the view which we have always taught here—that there are no distinct pathological varieties of phthisis; that the attempts so often made to subdivide the disease have no pathological basis. When it can be shown that the bacillus is present in certain cases of phthisis whilst it is absent in others, we shall be prepared to admit the existence of more than one form of the disease.

If some abnormal condition of the pulmonary apex necessarily precede any injurious influence of the bacillus, it becomes an interesting question as to how far such a condition can clinically be recognized. Although I cannot now support my belief by any carefully recorded facts, my impression is that cases are occasionally met with in which what is probably this pretubercular stage exists. In weakly individuals, in whom there are no marked lung symptoms, and in whom an examination of the chest is made, perhaps on account of cough which is really of gastric or pharyngeal origin, a few obscure, scattered crepitant *râles*, without any signs of consolidation, are sometimes discovered at one or both apices. In the course of a few days, after tonic treatment, these signs disappear, and there is no subsequent evidence of phthisical disease. In such cases, is not the condition of the pulmonary apices closely allied to that so common in the most dependent portions of the lungs of weakly subjects confined to bed, which gives rise to that transitory crepitation heard with the first few deep inspirations?

*Prognosis*.—It appears that there is a definite relation between the number of bacilli and the intensity of the phthisical process, so that the examination of the sputum may afford information of much prognostic value.

*Treatment*.—We now come to the most important part of our subject—that of treatment. What is the practical teaching of Koch's discovery with reference to the prevention and cure of phthisis? If our pathological conclusions be even only partially true, they clearly indicate, I think, the necessity of carefully distinguishing between the bacillus and the conditions which favor its influence, and of directing our treatment to both. We must endeavor to prevent the access of the organism, and, if possible, to destroy it after it has effected an entrance; and we must also strive to maintain a healthy condition of the pulmonary tissues, and thus prevent the occurrence of that tendency to apicial stagnation which appears to be such an important, if not essential, factor in the disease. The latter of these indications is, I believe, as important as the former; and it is, perhaps, rather in danger of being lost sight of in the very natural eagerness with which attention is now being directed towards the bacillus.

Firstly, then, with regard to the condition of the lung which favors the influence of the bacillus. Here it is only necessary to remark that, whatever promotes a vigorous state of health, will, by improving the condition of the blood, the nutrition of the vessels, the activity of the circulation, and the exercise of the respiratory function, tend to prevent that stagnation and transudation in the highest portions of the lungs, the etiological importance of which we have so especially insisted upon. The value of treatment which has for its object the fulfillment of these indications in the prevention of phthisis, it is, I believe, difficult to over-estimate; and its usefulness is almost equally valuable when the disease is established. I cannot but think that, in the meantime, such treatment promises better results than any attempts to attack the specific organisms.

Secondly, the tubercle-bacillus. The consideration of this naturally divides itself under two heads: (*a*) the prevention of its access, and (*b*) attempts to destroy it when the disease is developed.

(*a*) The prevention of the access of the bacillus. The present position of our knowledge appears to point to the desirability of adopting measures for the disinfection and destruction of the sputa of patients suffering from phthisis; and perhaps, also, of the alvine secretions, when there is any evidence of tuberculous disease of the bowel. It also raises the question as to how far it is desirable to allow individuals who are not consumptive, but who inherit a phthisical tendency, and especially when such individuals are out of health, to *intimately* associate with those who are suffering from the disease. If our pathology continues to move on the same lines, this subject may become one requiring the consideration of those who manage our hospitals.

(*b*) The destruction of the bacillus after the disease is established. Attempts to do this are made principally by means of antiseptic inhalations. This is the fashionable, though perhaps somewhat misdirected, therapeutics of the day. A respirator charged with some antiseptic, such as creosote or carbolic acid, is now being largely used in the treatment of phthisis. Although I should be very sorry to unfairly criticise such treatment, I cannot but think that the evidence that its usefulness is in any way dependent upon its destruction of the bacilli, or of any infective substance which they may originate, is wanting. It seems to me much more probable that such inhalations, when beneficial, are so mainly through the favorable influence which they exercise upon the mucous membrane and secretion ; and when, as is so often the case, they are combined with chloroform, they will also act as direct sedatives. What we want are cases of early and progressive phthisis in which antiseptic treatment alone, without adjuncts, is followed by marked improvement. When it can be shown, *e.g.*, that the pyrexia of early phthisis is reduced by such treatment, we shall have evidence pointing to the influence of the germicides upon the bacillus of considerable value. We are now making some observations in this direction, but, at present, with negative results. Whilst, therefore, I do not wish to be understood to discourage the treatment of phthisis by antiseptic inhalations, I think we must be careful as to the interpretation we put on our results. The treatment of phthisis and of other pulmonary diseases by means of a medicated atmosphere has been greatly stimulated by Koch's discovery. Such treatment has undoubtedly been too much neglected in the past, and its prosecution promises the best results. But, in the meantime, I think we have no evidence that we are able by such means to influence the tubercle bacillus; although, if Koch's investigations be true, the discovery of some agent which, by destroying it, will arrest its injurious influence, is obviously the greatest desideratum.—*British Medical Journal.*

